# Changes in transition times in ‘Ironman Hawaii’ between 1998 and 2013

**DOI:** 10.1186/2052-1847-6-37

**Published:** 2014-10-08

**Authors:** Christoph A Rüst, Thomas Rosemann, Romuald Lepers, Beat Knechtle

**Affiliations:** 1 Institute of Primary Care, University of Zurich, Zurich, Switzerland; 2 INSERM U1093, Faculty of Sport Sciences, University of Burgundy, Dijon, France; 3 Gesundheitszentrum St. Gallen, Vadianstrasse 26, 9001 St. Gallen, Switzerland

**Keywords:** Swimming, Cycling, Running, Sex difference, Endurance

## Abstract

**Background:**

Recent findings showed that elite Ironman triathletes competing in ‘Ironman Hawaii’ improved both split and overall race times. The present study investigated whether elite athletes also improved in transition time (*i.e.* time needed between disciplines for changing clothes and equipment).

**Methods:**

Changes in split times, overall race times and transition times (*i.e.* expressed in absolute and relative terms) in the annual fastest competing in ‘Ironman Hawaii’ were investigated using linear, non-linear and multi-level regression analyses. To detect a potential difference in transition times between different race distances, we compared transition times in ‘Ironman Hawaii’ to transition times in the World Championships ‘Ironman 70.3’ covering the half distance of the Ironman distance triathlon.

**Results:**

In ‘Ironman Hawaii’, transition times remained unchanged for the annual fastest women but increased linearly for the annual fastest men. For the annual ten fastest, transition times increased linearly for women and men in both absolute and relative terms. The sex difference in transition times remained unchanged for the annual fastest, but decreased linearly for the annual ten fastest. In ‘Ironman 70.3’, transition times remained unchanged for the annual fastest. For the annual ten fastest, transition times decreased linearly for both women and men in absolute and relative terms. The sex difference in transition times remained unchanged for both the annual fastest and the annual ten fastest. Transition times were faster in ‘Ironman 70.3’ for women in 2011 and for men in 2006, 2007, and 2010-2013. In relative terms, transition times were faster in ‘Ironman 70.3’compared to ‘Ironman Hawaii’ during 2006-2013. The sex difference in transition times remained unchanged.

**Conclusions:**

In ‘Ironman Hawaii’, transition times increased for both women and men whereas the sex difference decreased. In ‘Ironman 70.3’, transition times decreased for both women and men whereas the sex difference remained unchanged. Generally, transition times were slower in ‘Ironman Hawaii’ compared to ‘Ironman 70.3’.

## Background

Triathlon is a sport consisting of sequential swimming, cycling and running [1]. Triathlon races are mainly held on the short or Olympic distance (*i.e.* 1.5 km swimming, 40 km cycling, and 10 km running) [2] and the long distance, the Ironman distance (*i.e.* 3.8 km swimming, 180 km cycling, and 42.2 km running) [3]. Apart from these distances, also the sprint distance [4] and the half Ironman distance ‘Ironman 70.3’ [5] are of high popularity.

As one of the toughest and oldest long-distance triathlons in the world, the ‘Ironman Hawaii’ now serves as the World Championship for Ironman distance triathletes’ [6]. It has been recently shown that the annual top ten finishers have improved split and overall race times between 1983 and 2012, whilst also becoming older [7]. During the same period, the annual ten fastest women and men improved their swimming (only men), cycling, running, and overall race times [8]. In addition, the sex difference in overall race time decreased significantly from 15.2% to 11.3%. For the split disciplines, the sex difference remained unchanged for swimming (12.5 ± 3.7%) and cycling (12.5 ± 2.7%) but decreased for running from 13.5 ± 8.1% to 7.3 ± 2.9% [3,8].

During a triathlon, the triathletes have to change clothes and equipment between the swimming and the cycling part and then again between the cycling and the running part. These transition times between the three disciplines are part of the overall race time. As such, a triathlete’s ability to negotiate each transition quickly and effectively has been highlighted as an important factor for overall success in the event [9]. However, to our knowledge, no study has previously focused on the changes in the transition times over the years in Ironman triathletes. Overall race time could also be substantially improved by a reduction in transition times. Therefore, the purpose of the present study was to investigate the change in transition time in ‘Ironman Hawaii’ across years. Since both female and male triathletes have improved overall race times and split times during the last three decades, we hypothesized that both women and men also improved transition times across years. To investigate a potential difference in transition times between different race distances, we also compared transition times in ‘Ironman Hawaii’ to transition times in the World Championships ‘Ironman 70.3’ covering the half distance (1.9 km swimming, 90 km cycling and 21.1 km running) of the Ironman distance triathlon.

## Methods

### Ethics

All procedures used in the study met were approved by the Institutional Review Board of Kanton St. Gallen, Switzerland with a waiver of the requirement for informed consent of the participants given the fact that the study involved the analysis of publicly available data.

### Data sampling and data analysis

The data set for this study was obtained from the race website of the ‘Ironman World Championship’ [6]. All competitors who finished within the top ten in the ‘Ironman World Championship’ Hawaii between 1998 and 2013 were analyzed regarding their change in performance, the sex difference in performance and the transition times. Before 1998, transition times were not separately recorded in the race results. Therefore, data before 1998 could not be included into data analysis. To determine the sex difference in performance, overall race times and split times of the annual top and of the annual top ten women and men were taken from the race results of the organizer. Unfortunately, data from [6] do not allow to differentiate the two transition times (*i.e.* swimming to cycling and cycling to running) during the whole period 1998-2013. Therefore, absolute transition times were determined in absolute values by calculating the difference between overall race time and split times using the equation overall race times – (swimming times + cycling times + running times). Relative transition time was expressed as a percentage of overall race times using the equation 100/overall race times × transition times. The sex difference was calculated using the equation (times in women – times in men)/times in men × 100, where the sex difference was calculated for every pair of equally placed athletes (*e.g.* between female and male winner, between men and women 2^nd^ place, etc*.*) before calculating mean value and standard deviation of all pairs. In order to facilitate reading all sex differences were transformed to absolute values before analysing. In order to investigate a potential difference in transition times between different triathlon distances, we compared the transition times in ‘Ironman Hawaii’ to the transition times in the World Championships ‘Ironman 70.3’ covering the half distance of the Ironman triathlon (*i.e.* 1.9 km swimming, 90 km cycling and 21.1 km running) [10].

### Statistical analysis

Each set of data was tested for normal distribution and for homogeneity of variances prior to statistical analyses. Normal distribution was tested using a D’Agostino and Pearson omnibus normality test and homogeneity of variances was tested using a Levene’s test. Single and multi-level regression analyses were used to investigate changes in performance. A hierarchical regression model was used to avoid the impact of a cluster-effect on results in case one athlete finished more than once in the annual top or annual top ten. Since the change in sex difference in endurance performance is assumed to be non-linear [11], we additionally calculated the non-linear regression model that fits the data best. The result of the linear regression analysis was compared to the best-fit result of the non-linear analysis using Akaike’s Information Criteria (AIC) and F-test in order to show which model would be the most appropriate to explain the trend of the data. Split times in swimming, cycling, and running as well as overall race times and transition times in top ten women and men and differences in transition times between ‘Ironman Hawaii’ and ‘Ironman 70.3’ in top ten men and women were compared using multiple *t*-tests with Holm-Sidak correction for multiple comparisons. Statistical analyses were performed using IBM SPSS Statistics (Version 22, IBM SPSS, Chicago, IL, USA) and GraphPad Prism (Version 6.01, GraphPad Software, La Jolla, CA, USA). Significance was accepted at *P* < 0.05 (two-sided for *t*-tests). Data in the text and figures are given as mean ± standard deviation (SD).

## Results

### Changes in transition times in ‘Ironman Hawaii’

**Figure 1:**
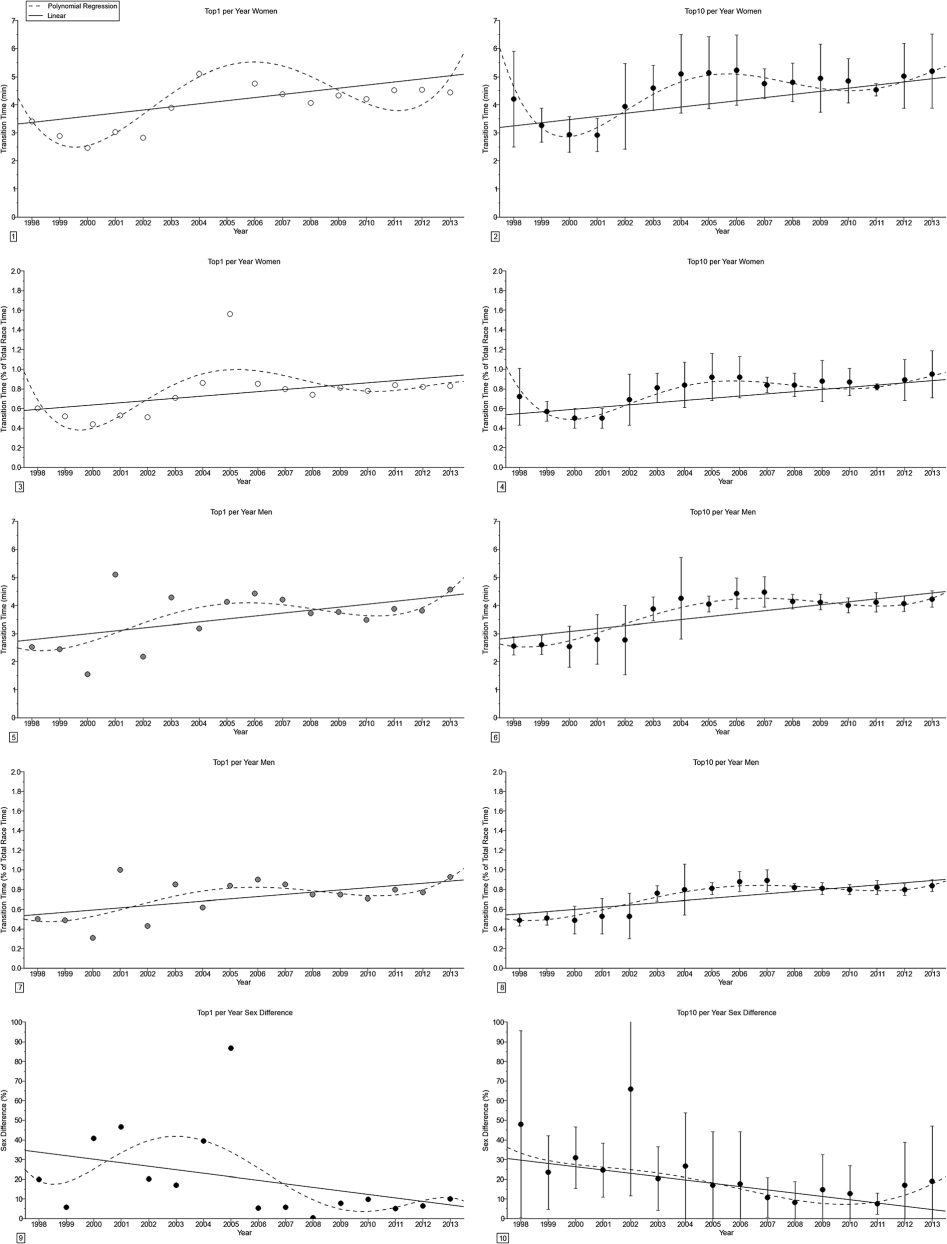
**Changes in transition times and sex difference in transition times in ‘Ironman Hawaii’ for the annual fastest and the annual ten fastest in absolute (*i.e. *min) and relative terms (*i.e. *expressed in % of overall race times) from 1998-2013. Panel 1:** Transition time for the annual fastest women. **Panel 2:** Transition time for the annual ten fastest women. **Panel 3:** Transition time in percent of overall race time for the annual fastest women. **Panel 4:** Transition time in percent of overall race time for the annual ten fastest women. **Panel 5:** Transition time for the annual fastest men. **Panel 6:** Transition time for the annual ten fastest men. **Panel 7:** Transition time in percent of overall race time for the annual fastest men. **Panel 8:** Transition time in percent of overall race time for the annual ten fastest men. **Panel 9:** Sex difference in transition time for the annual fastest. **Panel 10:** Sex difference in transition time for the annual ten fastest. Results are presented as mean ± SD for the annual ten fastest.

The transition times (Figure 1) remained unchanged across years for the annual fastest women but increased linearly for the annual fastest men in both absolute and relative terms (Table 1). For the annual ten fastest, the transition times increased linearly for both the annual ten fastest women and men in both absolute and relative terms. The sex difference in the transition times remained unchanged across years for the annual fastest triathletes, but decreased linearly for the annual ten fastest triathletes from 48.0 ± 47.6% (1998) to 18.9 ± 28.3% (2013).

**Table 1:** **Multi-level regression analyses for change in transition times (*i.e. *** **in absolute times and relative in % of overall race times) for the annual fastest and annual ten fastest across years after correction for multiple finishes in ‘Ironman Hawaii’**

	***β***	**SE ( *β *)**	**Stand. *β***	**T**	***P***
**Annual fastest women (absolute)**	0.111	0.073	0.378	1.528	0.149
**Annual fastest women (relative)**	0.023	0.013	0.420	1.734	0.105
**Annual fastest men (absolute)**	0.105	0.046	0.515	2.251	0.041
**Annual fastest men (relative)**	0.023	0.009	0.548	2.452	0.028
**Annual ten fastest women (absolute)**	0.118	0.020	0.427	5.931	<0.001
**Annual ten fastest women (relative)**	0.023	0.003	0.480	6.879	<0.001
**Annual ten fastest men (absolute)**	0.123	0.013	0.607	9.611	<0.001
**Annual ten fastest men (relative)**	0.026	0.002	0.646	10.625	<0.001
**Sex difference in the annual fastest**	-1.785	1.183	-0.374	-1.509	0.153
**Sex difference in the annual ten fastest**	-1.987	0.463	-0.323	-4.295	<0.001

### Changes in transition times in ‘Ironman 70.3’

**Figure 2:**
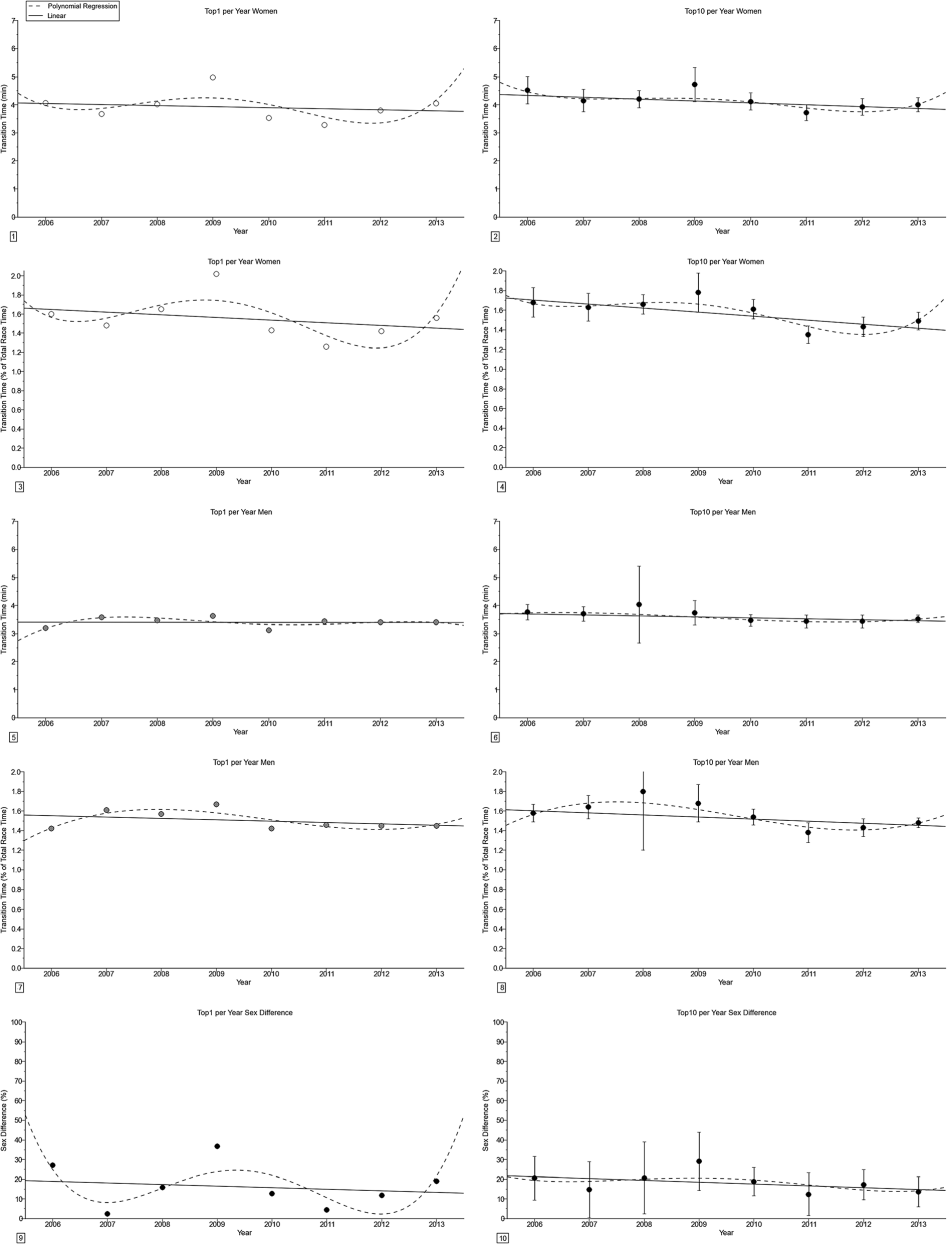
**Changes in transition times and sex difference in transition times in ‘Ironman 70.3’ for the annual fastest and the annual ten fastest in absolute (*i.e. *min) and relative terms (*i.e. *expressed in % of overall race times) from 2006-2013. Panel 1:** Transition time for the annual fastest women. **Panel 2:** Transition time for the annual ten fastest women. **Panel 3:** Transition time in percent of overall race time for the annual fastest women. **Panel 4:** Transition time in percent of overall race time for the annual ten fastest women. **Panel 5:** Transition time for the annual fastest men. **Panel 6:** Transition time for the annual ten fastest men. **Panel 7:** Transition time in percent of overall race time for the annual fastest men. **Panel 8:** Transition time in percent of overall race time for the annual ten fastest men. **Panel 9:** Sex difference in transition time for the annual fastest. **Panel 10:** Sex difference in transition time for the annual ten fastest. Results are presented as mean ± SD for the annual ten fastest.

In ‘Ironman 70.3’ (Figure 2), the transition times remained unchanged for the annual fastest in both absolute and relative terms (Table 2). For the annual ten fastest, transition times decreased linearly for both women and men in both absolute and relative terms. The sex difference in transition times remained unchanged across years for both the annual fastest and the annual ten fastest triathletes.

**Table 2:** **Multi-level regression analyses for change in transition times (*i.e. *** **in absolute times and relative in % of overall race times) for the annual fastest and annual ten fastest across years after correction for multiple finishes in ‘Ironman 70.3’**

	***β***	**SE ( *β *)**	**Stand. *β***	**T**	***P***
**Annual fastest women (absolute)**	-0.038	0.083	-0.182	-0.452	0.667
**Annual fastest women (relative)**	-0.028	0.036	-0.303	-0.779	0.465
**Annual fastest men (absolute)**	-0.001	0.028	-0.021	-0.050	0.962
**Annual fastest men (relative)**	-0.014	0.015	-0.350	-0.915	0.396
**Annual ten fastest women (absolute)**	-0.081	0.022	-0.392	-3.763	<0.001
**Annual ten fastest women (relative)**	-0.041	0.008	-0.524	-5.429	<0.001
**Annual ten fastest men (absolute)**	-0.061	0.026	-0.251	-2.292	0.025
**Annual ten fastest men (relative)**	-0.037	0.012	-0.327	-3.059	0.003
**Sex difference in the annual fastest**	-0.798	1.873	-0.171	-0.426	0.685
**Sex difference in the annual ten fastest**	-0.847	0.610	-0.155	-1.389	0.169

### Comparison of changes in transition times in ‘Ironman Hawaii’ and ‘Ironman 70.3’

**Figure 3:**
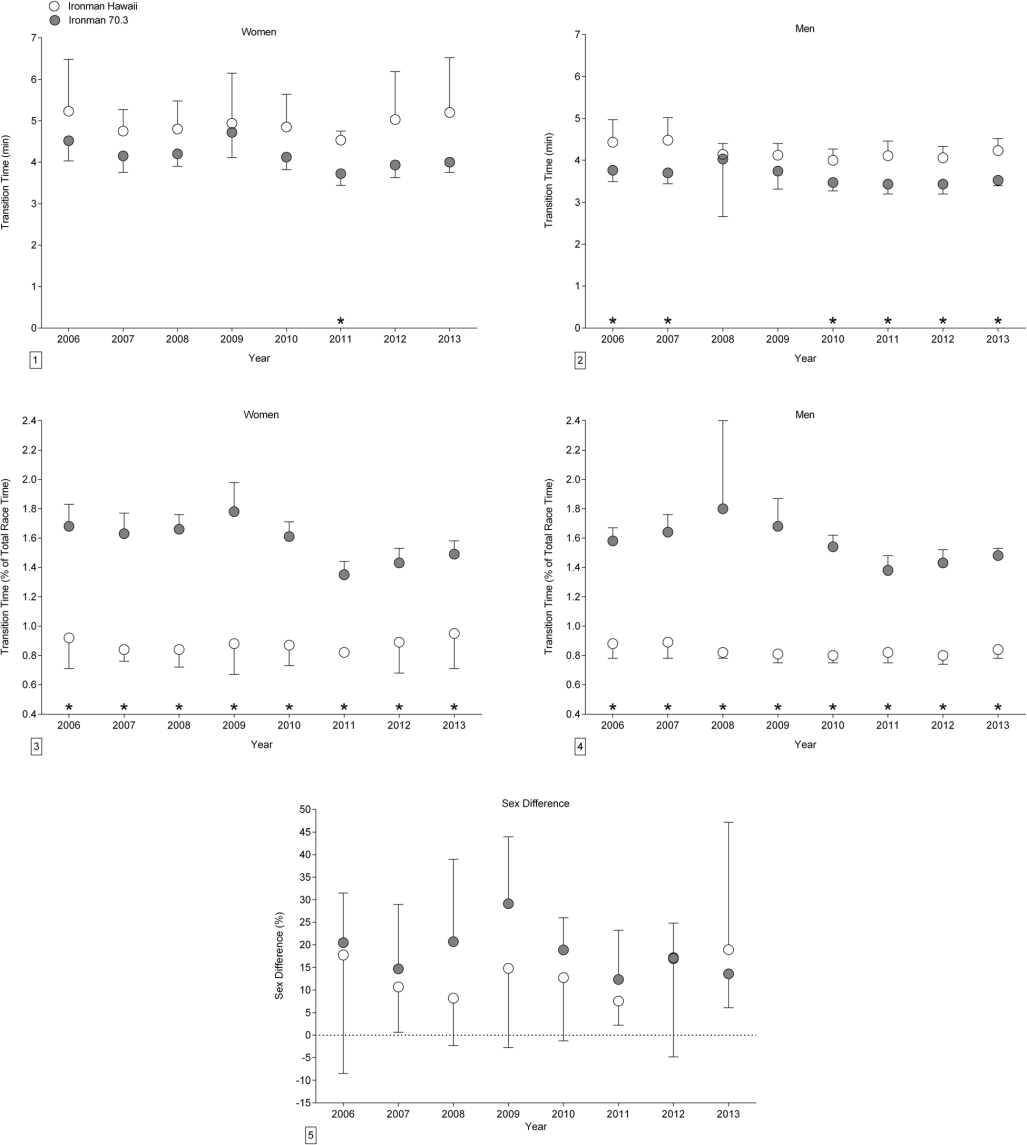
**Comparison of changes in transition times and sex difference in transition times between ‘Ironman Hawaii’ and ‘Ironman 70.3’ for the annual ten fastest in absolute (*i.e. *min) and relative values (*i.e. *expressed in % of overall race times) from 2006-2013. Panel 1:** Comparison for women in absolute times. **Panel 2:** Comparison for men in absolute times. **Panel 3:** Comparison for women in percent. **Panel 4:** Comparison for men in percent. **Panel 5:** Sex difference in transition times. Results are presented as mean ± SD.

When the changes in transition times across years between ‘Ironman Hawaii’ and ‘Ironman 70.3’ for the period 2006-2013 were compared in absolute terms for the annual ten fastest (Figure 3), transition times were faster in ‘Ironman 70.3’ for women in 2011 and for men in 2006, 2007, and 2010-2013. In relative terms, transition times were faster in ‘Ironman 70.3’ compared to ‘Ironman Hawaii’ during the whole period 2006-2013. The sex difference in transition times remained unchanged at 18.4 ± 11.5% during the whole period.

### Changes in split times, overall race times and sex difference in ‘Ironman Hawaii’

**Figure 4:**
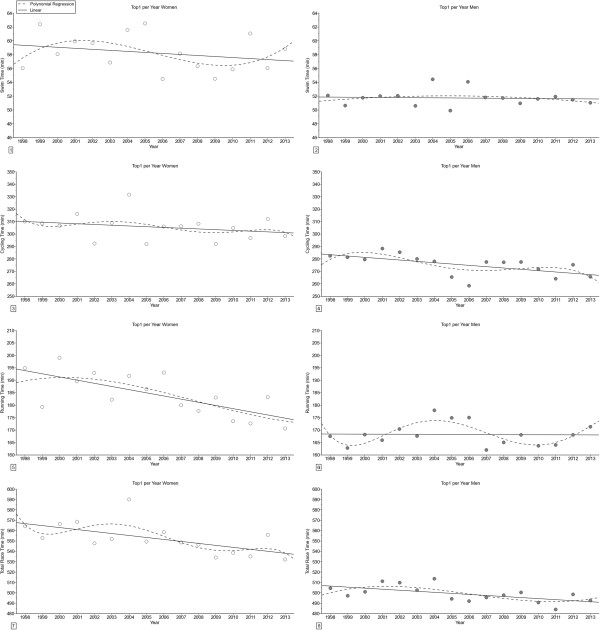
**Changes in split times and overall race times for the annual fastest women and men in ‘Ironman Hawaii’. Panel 1:** Swimming annual fastest women. **Panel 2:** Swimming annual fastest men. **Panel 3:** Cycling annual fastest women. **Panel 4:** Cycling annual fastest men. **Panel 5:** Running annual fastest women. **Panel 6:** Running annual fastest men. **Panel 7:** Total race time annual fastest women. **Panel 8:** Total race time annual fastest men.

**Figure 5:**
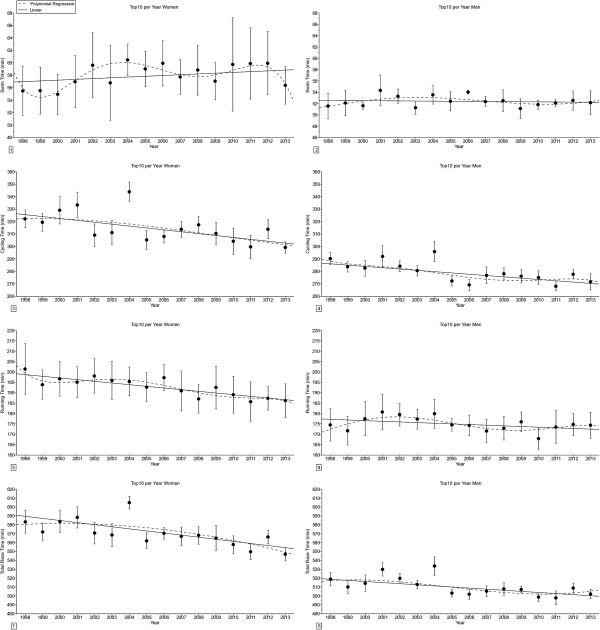
**Changes in split times and overall race times for the annual ten fastest women and men in ‘Ironman Hawaii’. Panel 1:** Swimming annual ten fastest women. **Panel 2:** Swimming annual ten fastest men. **Panel 3:** Cycling annual ten fastest women. **Panel 4:** Cycling annual ten fastest men. **Panel 5:** Running annual ten fastest women. **Panel 6:** Running annual ten fastest men. **Panel 7:** Total race time annual ten fastest women. **Panel 8:** Total race time annual ten fastest men. Results are presented as mean ± SD.

**Figure 6:**
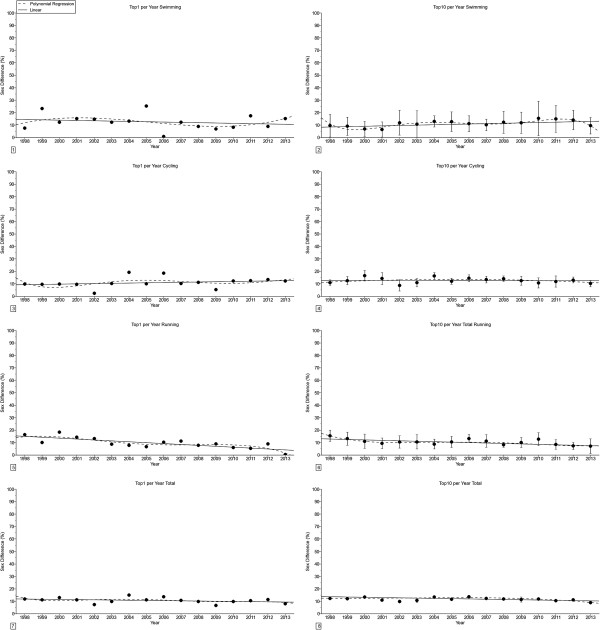
**Changes in sex difference in split times and overall race times in ‘Ironman Hawaii’ for the annual fastest and the annual ten fastest. Panel 1:** Sex difference in swimming annual fastest. **Panel 2:** Sex difference in swimming annual ten fastest. **Panel 3:** Sex difference in cycling annual fastest. **Panel 4:** Sex difference in cycling annual ten fastest. **Panel 5:** Sex difference in running annual fastest. **Panel 6:** Sex difference in running annual ten fastest. **Panel 7:** Sex difference in total race time annual fastest. **Panel 8:** Sex difference in total race time annual ten fastest. Results are presented as mean ± SD for the annual ten fastest.

For the annual fastest competitors (Figure 4), swimming split times remained unchanged, cycling split times decreased linearly in men, running split times decreased linearly in women and overall race times decreased linearly in both women and men (Table 3). For the annual ten fastest (Figure 5), swimming split times decreased linearly in women, cycling split times, running split times and overall race times decreased linearly in both women and men (Table 3). The sex difference in performance (Figure 6) decreased linearly in swimming for the annual ten fastest, in swimming for the annual fastest and the annual ten fastest and for overall race in the annual ten fastest (Table 4). For cycling, the sex difference remained unchanged for both the annual fastest and the annual ten fastest.

**Table 3:** Multi-level regression analyses for changes in split and overall race times for the annual fastest and annual ten fastest women and men across years after correction for multiple finishes

**Times**	***β***	**SE ( *β *)**	**Stand. *β***	**T**	***P***
**Annual fastest women**					
**Swimming**	-0.148	0.146	-0.263	-1.018	0.326
**Cycling**	-0.595	0.548	-0.279	-1.086	0.296
**Running**	-1.273	0.340	-0.708	-3.747	0.002
**Overall**	-1.905	0.671	-0.605	-2.841	0.013
**Annual fastest men**					
**Swimming**	-0.018	0.065	-0.074	-0.276	0.787
**Cycling**	-1.069	0.369	-0.613	-2.900	0.012
**Running**	-0.024	0.260	-0.025	-0.092	0.928
**Overall**	-1.006	0.359	-0.599	-2.802	0.014
**Annual ten fastest women**					
**Swimming**	0.200	0.076	0.203	2.610	0.010
**Cycling**	-1.544	0.214	-0.498	-7.223	<0.001
**Running**	-0.860	0.143	-0.431	-6.011	<0.001
**Overall**	-2.087	0.246	-0.560	-8.498	<0.001
**Annual ten fastest men**					
**Swimming**	-0.022	0.031	-0.055	-0.695	0.488
**Cycling**	-1.180	0.135	-0.572	-8.760	<0.001
**Running**	-0.285	0.115	-0.194	-2.480	0.014
**Overall**	-1.364	0.175	-0.526	-7.778	<0.001

**Table 4:** Multi-level regression analyses for changes in sex difference in split and overall race times for the annual fastest and annual ten fastest women and men across years after correction for multiple finishes

**Times**	***β***	**SE ( *β *)**	**Stand. *β***	**T**	***P***
**Annual fastest**					
**Swimming**	-0.253	0.336	-0.197	-0.753	0.464
**Cycling**	0.213	0.222	0.248	0.959	0.354
**Running**	-0.725	0.155	-0.781	-4.673	<0.001
**Overall**	-0.159	0.113	-0.352	-1.405	0.182
**Annual ten fastest**					
**Swimming**	0.342	0.142	0.189	2.416	0.017
**Cycling**	-0.079	0.066	-0.095	-1.205	0.230
**Running**	-0.302	0.081	-0.286	-3.747	<0.001
**Overall**	-0.112	0.026	-0.324	-4.299	<0.001

## Discussion

This study investigated the changes in transition times in ‘Ironman Hawaii’ across years between 1998 and 2013. It was hypothesized that both elite women and men would improve transition times across years since they also improved split and overall race times across years. To investigate a potential difference in transition times between different race distances, transition times in ‘Ironman Hawaii’ were compared to transition times in the World Championships ‘Ironman 70.3’ covering the half distance of the Ironman triathlon. The main findings were that (*i*) in ‘Ironman Hawaii’, transition times increased in both women and men whereas the sex difference in transition times decreased, (*ii*) in ‘Ironman 70.3’, transition times decreased in both women and men whereas the sex difference remained unchanged, and (*iii*), transition times were slower in ‘Ironman Hawaii’ compared to ‘Ironman 70.3’.

### Changes in performance and transition times across years

In ‘Ironman Hawaii’, swimming split times decreased linearly in women, cycling split times, running split times and overall race times decreased linearly in both women and men for the annual ten fastest. The transition times increased linearly for both the annual ten fastest women and men in both absolute and relative terms. Generally, elite Ironman triathletes competing in ‘Ironman Hawaii’ improved performance in both split and overall race times [3]. A recent study investigating the change in performance across years in elite finishers in ‘Ironman Hawaii’ from 1983 to 2012 using only linear regression analyses showed similar findings where the annual ten fastest women and men improved their swimming (only men), cycling, running, and overall race times [7].

The new finding in the present study was that transition times increased across years, although it was hypothesized that transition times would decrease since both women and men improved split and overall race times. A potential explanation for this unexpected finding might be that the transition area has changed across years. The distances between the exit out of the water to the cycling area and the distance between the finish of the cycling to the start of the running might have increased and the athletes might have needed to run longer to change the discipline. A potential change in the transition area could be due to the increasing number of participants in ‘Ironman Hawaii’ in the last years. Another explanation could be that the athletes focused more on the performance in swimming, cycling and running [12] and were not aware that overall race time could also be substantially improved by a reduction in transition times. And a further explanation could be the different time periods investigated in the different studies.

### Differences in transition times between ‘Ironman 70.3’ and ‘Ironman Hawaii’

The comparison of the transition times between ‘Ironman 70.3’ and ‘Ironman 70.3’ showed that transition times decreased in both absolute and relative terms in the shorter race distance ‘Ironman 70.3’ whereas in the longer race distance ‘Ironman Hawaii’ the transition times increased also in both absolute and relative terms. When the transition times were compared between ‘Ironman 70.3’ and ‘Ironman Hawaii’, athletes changed faster in the shorter race ‘Ironman 70.3’ than in the longer race ‘Ironman Hawaii’. A potential explanation for this finding could be that the shorter race is faster in both the split and the transition times than the longer race. A further explanation could be that the transition area is smaller in ‘Ironman 70.3’ compared to ‘Ironman Hawaii’ due to the different numbers of participants. During the years, athlete may also have improved their technique to change faster in the transition area [13-15]. Maybe the athletes are more exhausted in the full Ironman than in the half Ironman after a split discipline when they enter the transition area and are therefore slower in the transition in the full Ironman distance compared to the half Ironman distance.

Other explanations could be different transition areas in the different races and the different location of the races. Apart from changes in the transition area across years, the World Championship ‘Ironman Hawaii’ was held during the investigated period at the same place whereas the World Championship ‘Ironman 70.3’ was held in 2006-2010 in Clearwater, Florida, USA, and in 2011-2013 in Henderson, Nevada, USA. The two different race courts might also have different transition areas.

### Limitations and implications for future research

A limitation in this study is that the transition area may have changed during this period and therefore the absolute time an athlete spent between two disciplines may have be different from year to year. However, the transition times were expressed here as absolute values and as relative values as a percentage of overall race time. When the transition times changed due to changes of the transition area, overall race time changed accordingly. A further limitation is that transition times between swimming and cycling and between cycling and running were not separated but unfortunately transition times between the disciplines were provided only in recent years. The age of the athletes was not included. It has recently been shown that sex differences in overall race times differed between age groups in sprint, Olympic distance, and half Ironman triathlon [16]. Since the sex difference in long-distance triathlon increased with increasing race distance [17], future studies should investigate the sex difference in transition times for different triathlon distances. In addition, the present study focused on elite triathletes only, it would interesting to analyse (*i*) the sex difference in transition times for age groups triathletes and (*ii*) the age-related change in transition times. It is also worth considering in future research what would constitute a practically meaningful change in ‘Ironman’ and ‘Ironman 70.3’ transition times in the context of transition times alone and in relation to overall performance. For example, when the typical within-athlete variability is not available, the average time difference between each of the top ten finishing positions would give an idea of a worthwhile enhancement in performance times, and, more importantly, in transition times.

## Conclusions

In summary, transition times increased for both women and men in ‘Ironman Hawaii’ whereas the sex difference in transition times decreased. In ‘Ironman 70.3’, transition times decreased for both women and men whereas the sex difference in transition times remained unchanged. Generally, transition times were slower in ‘Ironman Hawaii’ compared to ‘Ironman 70.3’. Top ten finishers in ‘Ironman Hawaii’ might further improve their overall race times by reducing their transition times.

## Competing interests

The authors declare that they have no competing interests.

## Authors’ contributions

CR performed the statistical analyses and drafted the manuscript, BK collected the data and helped in drafting the manuscript, TR and RL helped in drafting the manuscript. All authors read and approved the final manuscript.

## Pre-publication history

The pre-publication history for this paper can be accessed here:

http://www.biomedcentral.com/2052-1847/6/37/prepub
